# New tools to prevent cancer growth and spread: a ‘Clever’ approach

**DOI:** 10.1038/s41416-020-0953-0

**Published:** 2020-06-29

**Authors:** Maija Hollmén, Carlos R. Figueiredo, Sirpa Jalkanen

**Affiliations:** MediCity Research Laboratory and Institute of Biomedicine, University of Turku, Turku, Finland

**Keywords:** Cancer immunotherapy, Translational research

## Abstract

Clever-1 (also known as Stabilin-1 and FEEL-1) is a scavenger receptor expressed on lymphatic endothelial cells, sinusoidal endothelial cells and immunosuppressive monocytes and macrophages. Its role in cancer growth and spread first became evident in *Stab1*^–/–^ knockout mice, which have smaller primary tumours and metastases. Subsequent studies in mice and humans have shown that immunotherapeutic blockade of Clever-1 can activate T-cell responses, and that this response is mainly mediated by a phenotypic change in macrophages and monocytes from immunosuppressive to pro-inflammatory following Clever-1 inhibition. Analyses of human cancer cohorts have revealed marked associations between the number of Clever-1-positive macrophages and patient outcome. As hardly any reports to date have addressed the role of Clever-1 in immunotherapy resistance and T-cell dysfunction, we performed data mining using several published cancer cohorts, and observed a remarkable correlation between Clever-1 positivity and resistance to immune checkpoint therapies. This result provides impetus and potential for the ongoing clinical trial targeting Clever-1 in solid tumours, which has so far shown a shift towards immune activation when a particular epitope of Clever-1 is blocked.

## Background

Immunotherapy is not a new approach for the treatment of cancer: as early as the 1890s, William Coley, the father of immunotherapy, observed beneficial effects in certain cancer patients^1^ after injecting them with a mixture of *Streptococcus pyogenes* and *Serratia marcescens* (Coley’s toxin) to boost the immune system. During the last 9 years, we have witnessed the power of immunotherapy when targeting specific molecules, such as cytotoxic T-lymphocyte-associated protein 4 (CTLA-4), programmed cell death-1 (PD-1) and programmed death-ligand 1 (PD-L1), that control the magnitude of immune responses. Moreover, our understanding of the immune landscape of cancer has improved, and we now appreciate that this immune landscape might be equally as important as, if not more important than, other factors, such as tumour type, in predicting the survival of patients.^2^ However, despite excellent progress in the development of therapies targeting immune checkpoint molecules, durable effects are observed only in a subset of cancer patients while being ineffective in the majority of patients.^3^ Currently, it is not well known, which parameters are finally decisive in the effectiveness of the immunotherapies, but many things besides the tumour type, such as the tumour burden,^4,5^ mismatch repair deficiency/microsatellite instability,^6^ heterozygosity at HLA-I loci,^7^ gut microbiota^8^ and age of the patient,^9^ contribute to the outcome.

Consequently, new therapeutic targets are actively being sought, and more than 3000 clinical trials with agents that target the immune system to treat cancer are ongoing (https://clinicaltrials.gov). One such target is Clever-1 (also known as Stabilin-1 and FEEL-1), a large glycoprotein receptor that is expressed on the surface of lymphatic endothelial cells, sinusoidal endothelial cells and immunosuppressive macrophages and monocytes, and which is involved in scavenging, angiogenesis and cell adhesion.^10–12^ As a scavenger receptor, Clever-1 is known to bind and endocytose a wide range of ligands, from lipoproteins to carbohydrates, and, therefore, plays an important role in tissue homoeostasis and remodelling—hence its expression is at high levels in liver sinusoidal endothelial cells, which are responsible for clearing by-products or degradation intermediates of macromolecule turnover from the circulation, and in macrophages, in which Clever-1 is involved in receptor-mediated endocytosis, intracellular sorting and recycling.^13,14^ Clever-1 is expressed both on the afferent and efferent lymphatics, where it mediates the migration of incoming lymphocytes into the draining lymph nodes and also their exit from the nodes, respectively.^15^ Based on ex vivo adhesion assays using human lymph nodes and lymphatic endothelial cells, Clever-1 is involved at least in lymphocyte binding to the lymphatic endothelium and subsequent transmigration.^12,16^ Clever-1 is absent from normal flat-walled venules, but can be induced on the vasculature at sites of inflammation and in the tumour.^17,18^ Given the presence of Clever-1 in type 2 macrophages and lymphatic endothelial cells, it is perhaps not surprising that this protein has been revealed to have a role in cancer, with mice lacking Clever-1 shown to have smaller primary and metastatic tumours than control mice.^18^ In this review, we describe the unique characteristics of Clever-1 and its potential as a target for cancer therapy.

## An overview of Clever-1 structure and function

**Fig. 1:**
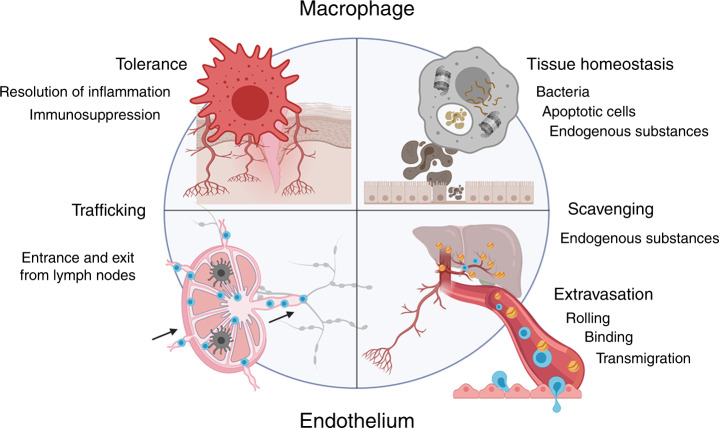
A schematic representation of multiple functions of Clever-1. Scavenging and trafficking are shared functions for both macrophage and endothelial cell Clever-1. Although macrophage Clever-1 is an important scavenger, it also mediates macrophage binding to the endothelium and Clever-1 on the endothelium, besides mediating leukocyte binding that is also able to scavenge.

Clever-1 (common lymphatic endothelial and vascular endothelial receptor-1)^12^ was identified by our group during a search for molecules responsible for cell trafficking within lymphatic vessels; simultaneously, Politz et al.^11^ reported the DNA sequence of the MS-1 antigen present on alternatively activated macrophages and spleen sinusoidal endothelial cells,^20^ which they named Stabilin-1, and Adachi and Tsujimoto^10^ reported a molecule, which they named FEEL-1 (fasciclin, epidermal growth factor [EGF]-like, laminin-type EGF-like and link domain-containing scavenger receptor-1), that was able to scavenge bacteria and bind to acetylated low-density lipoprotein (ac-LDL). Sequencing of the gene encoding Clever-1 revealed it to be a 270–280-kDa type 1 transmembrane protein with a multidomain nature, containing clusters of EGF-like domains, seven fasciclin domains and one X-link domain, and to be a close relative of Stabilin-2, a large transmembrane receptor that has roles in clearance of necrotic cells, internalisation of von Willebrand factor–Factor VIII complex and in lymphocyte binding to liver endothelium.^19,21,22^ Moreover, Stabilin-2 binds to hyaluronan that Clever-1 does not do, although it contains the X-link domain, located close to the transmembrane region of the molecule.^11,16^ Clever-1 contains 69 exons, which creates several possibilities for alternative splicing, and at least two isoforms of Clever-1 have been detected at the protein level.^12,13^ Clever-1 rapidly cycles between the plasma membrane and endosomal compartments, and its surface expression is often much lower than its intracellular one,^23^ observations that are consistent with its role as a scavenger receptor. Diverse functions of Clever-1 are depicted in Fig. 1.

## Clever-1 in homoeostasis

On macrophages, Clever-1 functions as a scavenger for Gram-positive and Gram-negative bacteria, several endogenous proteins such as ac-LDL, placental lactogen, secreted protein acidic and rich in cysteine (SPARC) and advanced glycation end products; Clever-1 on macrophages can also bind to phosphatidylserine residues on the surface of apoptotic cells and thus clear these dying cells to maintain homoeostasis.^10,24–27^ Moreover, Clever-1-positive macrophages can use it for the binding to vascular endothelium.^28^

As mentioned above, the scavenger role of Clever-1 is required for clearing degradation products from the circulation.^14^ Ex vivo studies using human vascular and lymphatic endothelial cells have shown that Clever-1 is involved in lymphocyte transmigration.^16,29^ Furthermore, intracellular crawling of lymphocytes in human primary hepatic sinusoidal endothelial cells from one cell into another has been demonstrated to be partially dependent on Clever-1.^30,31^ Additional in vivo studies using mouse and rabbit models have demonstrated that Clever-1 mediates leukocyte and cancer cell trafficking via the lymphatic system into the draining lymph nodes and lymphocyte homing to the spleen.^18,32,33^ Besides being able to directly bind lymphocytes, Clever-1 regulates its endothelial microenvironment. For example, the C–X–C motif chemokine ligand 13 (CXCL13) chemokine, attracting B cells on the splenic red pulp vessels, is markedly downregulated at the mRNA and protein level in the absence of Clever-1. This leads to impaired homing of the B cells into the spleen.^33^ Dendritic cell transmigration from the skin lymphatics into the draining lymph nodes is also compromised in the absence of Clever-1. However, at the same time, the lymphatic endothelial cells are less tolerogenic and can support adaptive immune responses with lower numbers of migratory dendritic cells.^34^

Clever-1 full-knockout mice and mice conditionally lacking Clever-1 have been created,^14,18^ and are seemingly healthy and reproduce normally if not challenged by any means. Although single knockouts of Clever-1 do not display drastic problems in degrading endogenous substances compared with double knockouts (*Stab1*^−/−^*Stab2*^−/−^), they do show a somewhat increased amount of perisinusoidal fibres,^14^ which is consistent with the role of Clever-1 expressed in liver sinusoidal endothelial cells in the hepatic clearance of potentially noxious agents. Notably, however, hepatic fibrosis was accelerated and its resolution was delayed during chronic liver injury in *Stab1*^*–/–*^ mice,^35^ which points to the involvement of macrophages. Clever-1 on macrophages is therefore thought to control the tissue microenvironment during liver injury and healing, suggesting that Stabilin-2 can to some extent compensate for the absence of Clever-1 on endothelial cells but not on macrophages in single-knockout mice. This conclusion can be made by looking at the expression pattern of *Stab1* and *Stab2* in mouse tissue macrophages (http://rstats.immgen.org/Skyline/skyline.html). The most notable expression of *Stab1* can be found in adipose tissue macrophages, microglia and to some extent in peritoneal macrophages, whereas *Stab2* is highly expressed only by red pulp macrophages in the spleen. In humans, both the *STAB1*-positive breast-tissue-resident macrophages and monocyte-recruited tumour-associated macrophages (TAMs) are *STAB2* negative.^36^ Clever-1-deficient mice also have higher immunoglobulin levels and respond more vigorously to immunisations by producing higher amounts of antibodies.^37^ These observations are thought to be due to the higher production of tumour necrosis factor (TNF)-α by Clever-1-deficient macrophages as TNF-α is known to promote immunoglobulin production.^37^ This observation suggests that Clever-1 normally functions as an immunosuppressive molecule, and that its lack leads to immune activation.

## Clever-1 in immunosuppression and cancer

A remarkable similarity exists between pregnancy and cancer with regard to the immunosuppressive milieu that prevails in the placenta to tolerate the growing foetus and in the surrounding tissue to support tumour development. A common feature of both situations is the high abundance of Clever-1-positive macrophages, which support the formation of an immunosuppressive tissue microenvironment.^28^ During pre-eclampsia, in which an abnormal pro-inflammatory reaction occurs in the placenta, Clever-1 expression is downregulated on placental macrophages and peripheral monocytes, suggesting a link between Clever-1 expression and the level of immunosuppression.^38^ In the context of cancer, Clever-1 was first described to be expressed on tumour lymphatic vessels in breast and head and neck cancer, where it is thought to facilitate metastatic spreading of cancer cells to regional lymph nodes via adhesion to intratumoural lymphatic vessels.^39^ This lymphatic vessel involvement as a prognostic factor for poor survival was subsequently demonstrated in patients with colorectal carcinoma (CRC).^40^ Importantly, those patients with a high number of both peritumoral and intratumoural Clever-1-positive macrophages in the advanced stages of CRC also have shorter disease-specific survival. In particular, a shift in the balance towards type 2 (M2) macrophages with an immunosuppressive phenotype as a proportion of the total number of macrophages was a decisive factor in determining tumour behaviour and prognosis. It seems that the expression of Clever-1 on TAMs might also serve as an effective marker for disease outcome as, in urothelial bladder cancer, for example, patients with a high abundance of Clever-1-positive TAMs have a higher mortality after transurethral resection.^41^ Also in urothelial bladder cancer, Clever-1 expression on TAMs but not on lymphatic endothelial cells is associated with a poor response to neoadjuvant chemotherapy and poorer overall survival.^42^ Similarly, in another cohort of patients with bladder cancer, intratumoural Clever-1 TAM density is an independent prognostic factor for poor overall survival,^43^ and increases the risk of recurrence in patients with oral cavity cancers.^44^

Gene expression analyses using multiple datasets have also identified high *STAB1* expression as a prognostic factor for poor outcome in patients with cytogenetically normal acute myeloid leukaemia (CN-AML).^45^ The same study shows that not only does Clever-1 suppression in AML cell lines (KG-1 and NB4) inhibit their proliferation, but it also makes them more sensitive to the BCL2 inhibitor, venetoclax, resulting in increased apoptosis of the cells. Apart from AML, no other studies have reported Clever-1 expression in cancer cells, per se, although one study shows that breast cancer cells undergoing epithelial–mesenchymal transition (EMT) induced by transforming growth factor (TGF)-β acquire myeloid-specific gene expression, including Clever-1, which confers invasive and migratory capabilities.^46^

## Potential mechanism(s) of action of Clever-1 in tumour growth

Because of the endothelial and myeloid expression of Clever-1, as well as its multiple functions, the precise mechanism by which Clever-1 promotes tumour growth has been challenging to assess. By developing cell-specific Clever-1-knockout mice using Tie2 (to target the blood endothelium) and LyzM (to target the myeloid lineage) promoters, Karikoski and colleagues have shown that tumour-induced Clever-1 expression in both endothelial cells and macrophage populations is needed to support the primary growth of melanoma, but that metastatic spread is mostly regulated by lymphatic endothelial cell Clever-1.^18^ Immunotherapeutic targeting of Clever-1 in a mouse melanoma model, aberrantly expressing Clever-1 on the tumour vasculature, leads to diminished expression of vascular E- and P selectin, and decreased accumulation of type 2 macrophages and regulatory T cells in the tumours.^18^

In a follow-up study using the same knockout mice in conjunction with bone marrow chimaeras and cell-depletion experiments with anti-CD8 and anti-(colony-stimulating factor-1 receptor) (CSF-1R), we demonstrated that primary tumour growth in syngeneic lung (LLC), lymphoma (EL-4), breast (4T1) and colon (CT26) tumour models was almost entirely regulated by Clever-1 expression in macrophages.^47^ When Clever-1 was immunotherapeutically blocked, TAMs showed increased pro-inflammatory cytokine secretion and antigen presentation, which supported cytotoxic CD8 T-cell activation and antitumour immunity.^47^ Similarly, downregulation of Clever-1 with small-interfering RNA (siRNA) in human monocytes resulted in the upregulation of several genes that regulate pro-inflammatory mediators, such as the chemokine CXCL13, the cytokine oncostatin M (OSM), serum amyloid A (SAA2), higher secretion of TNF-α and dampening of antigen-specific (timothy grass extract) T helper 2 (T_H_2) cell responses,^38^ consistent with an immunosuppressive role for Clever-1. We have also demonstrated that human placenta-purified Clever-1 can directly bind both B- and CD8 T cells, and to a lesser extent CD4 T cells from mouse spleen^33^ and human peripheral blood.^48^ Thus, it is possible that soluble Clever-1 can directly inhibit lymphocyte activation or transmigration by binding a yet unknown ligand on B- and CD8 T cells, and does not need immunosuppressive support from the type 2 macrophage. In fact, Clever-1 can be found as a soluble form in human blood and lymph where it could possibly interact with lymphocytes to regulate their functions.^48^

Clever-1 constantly recirculates from the cell membrane to the endosomal compartments transporting its cargo for degradation. This requires efficient phago-lysosomal fusion and acidification of vesicles. Studies performed on dendritic cells have shown that efficient cross-presentation of internalised or phagocytosed antigens is managed by restricting antigen degradation.^49^ Dendritic cells express low levels of lysosomal enzymes and prevent the assembly of the V-ATPase complex (the main proton transporter in lysosomes), which results in higher lysosomal pH.^50^ It can be speculated that the inhibition of Clever-1 alters the phago-lysosomal fusion kinetics of macrophages, leading to more effective peptide loading on MHC I and increased cross-presentation of antigens to CD8 T cells.^51^ The change in the molecular composition of the lysosomes would further support the mammalian target of rapamycin (mTOR) signalling and increased inflammatory cytokine secretion that we have seen in Clever-1-deficient mouse macrophages.^47^

Indeed, one complicating factor in Clever-1 biology is the context-dependent abundance and function of various Clever-1 ligands in the tumour microenvironment (TME) that might influence the antitumour effects observed when blocking Clever-1. As such, the contribution of macrophage-expressed Clever-1 to tumour progression has also been suggested to result from the clearance of extracellular tumour-growth-inhibiting factors, which ultimately leads to a change in the composition of the TME that favours tumour growth.^52^ Although high levels of SPARC are associated with a highly aggressive tumour type in some cancers, this matricellular glycoprotein—a Clever-1 ligand—can also function as a tumour suppressor in others.^53^ SPARC inhibits growth, angiogenesis and invasion in various cancer cells, which supports the idea that blocking Clever-1 scavenging as a means of increasing SPARC in the TME might be clinically beneficial in certain tumour types. In fact, inhibiting Clever-1-mediated SPARC degradation in neuroblastoma increases chemosensitivity of the tumour to nab-paclitaxel.^54^ Another Clever-1 ligand, stabilin-1-interacting chitinase-like protein (SI-CLP), inhibits tumour growth in a mouse breast cancer model by blocking the cytokine-induced recruitment of TAMs and altering the cell composition of the TME.^55^ As the expression of SI-CLP has been reported to be markedly downregulated or absent in some human breast cancers,^55^ targeting Clever-1 might again prove a worthwhile strategy.

Overall, the main assumption is that blocking Clever-1 on macrophages releases their antigen-presenting capability and potential to respond to pathogen- or danger-associated molecular patterns within tissues. There are several other macrophage-targeting strategies in clinical trials that act to stimulate the anti-tumoural properties of TAMs. Several of them promote Toll-like receptor (TLR) and CD40 activation, induce interferon production (stimulator of interferon genes, STING agonists), inhibit the immunosuppressive adenosine pathway (anti-CD73, anti-adenosine A2A receptor [A2AR]) or promote phagocytosis of cancer cells by inhibiting the CD47-signal regulatory protein α (SIRPα) ‘don’t eat me' signalling axis.^56^ Only few of these clinical programmes have yielded promising results and many have been terminated due to toxicities. This has been mainly caused by feedback mechanisms that counter-regulate the desired effects of the drug, or are related to unspecific activities of the drug that increases side effects.^56^ We consider that Clever-1 is likely to induce immune switching in TAMs, but works in a different way than, for example, the selective class IIa histone deacetylase (HDAC) inhibitors^57^ since we do not see impaired TAM differentiation (CD206 expression) in the presence of anti-Clever-1.^18^ As we do see increased and prolonged nuclear factor κB (NF-κB) phosphorylation in lipopolysaccharide-treated Clever-1-deficient mouse macrophages,^47^ supposedly Clever-1 has somewhat similar downstream effects with phosphoinositide 3-kinase γ (PI3Kγ) signalling. Pharmacological inhibition of PI3Kγ, a key regulator of pro-tumoural macrophages, activates NF-κB and converts TAMs into a pro-inflammatory state subsequently inhibiting tumour growth.^58^

## Immunotherapeutic targeting of Clever-1

Most of the antibodies targeting Clever-1 reported in the literature have been produced by the Goerdt and Jalkanen groups. The mStab1.26 (IgG1) antibody, which recognises mouse Clever-1, was generated by immunising Clever-1-knockout mice with the amino-terminal part of Clever-1,^59^ and has been widely used in functional in vivo studies because of its antagonistic similarities with the 3-372 (IgG1) mouse anti-(human Clever-1) antibody generated by our group.^12,39^ Both anti-mouse (mStab1.26) and anti-human (3-372) Clever-1 antibodies inhibit leukocyte adhesion to tumour vessels in ex vivo adhesion assays.^18,39^ Moreover, treatment with the mStab1.26 antibody leads to activation of antitumour immune responses^47^ and apoptosis of cancer cells in syngeneic mouse tumour models.^18^ The mStab1.26 antibody has not been shown to deplete TAMs or downregulate Clever-1 expression on TAMs. Thus, the mode of action is considered to result from impaired or re-routed trafficking of the receptor and its ligands within the cell. In fact, the 3-372 antibody can revert ac-LDL scavenging-related suppression of CCL3 secretion in human monocytes by inhibiting the scavenging of ac-LDL.^35^ Hence, it does not inhibit phagocytosis of *Staphylococcus aureus* or apoptotic cells (unpublished observations). The increased pro-inflammatory phenotype gained by targeting Clever-1 on human monocytes with 3-372 results in increased antigen-specific CD4 T_H_1 T-cell responses to tetanus toxoid,^38^ similar to the observed increase in CD8^+^ T-cell proliferation in lung tumours after anti-Clever-1 targeting with the mouse mStab1.26 antibody.^47^ However, despite having overlapping functions on immune activation and leukocyte adhesion, the two antibodies cannot be directly compared since the Clever-1 epitope recognised by 3-372 is not shared with mouse Clever-1.^60^ Also, worth noting is that other antibodies binding human Clever-1 with non-overlapping epitopes, such as 9–11 (rat IgG2a), do not produce similar pro-inflammatory changes in monocytes and macrophages as seen with 3-372.^51^ This is possibly due to the multifunctional properties and various ligand-binding sites on Clever-1.

The potential benefits of targeting Clever-1 to overcome the immunosuppressive TME have led to the development of Clevegen, a humanised anti-Clever-1 IgG4 antibody originating from 3-372, which entered clinical trials in December 2018. The MATINS (Macrophage Antibody to FP-1305 To INhibit immune Suppression, NCT03733990) study is the first-in-human open-label Phase 1/2 adaptive clinical trial in selected metastatic or inoperable solid tumours to investigate the safety and efficacy of Clevegen. The selected tumours include cutaneous and uveal melanoma, hepatobiliary, pancreatic, ovarian, oestrogen-receptor-positive breast, colorectal, gastric, gallbladder cancer and cholangiocarcinoma, all of which are known to contain high amounts of Clever-1-positive macrophages. Early results from the dose-escalation part of the study, which includes 11 treated patients, show promising tolerability, clinical antitumour activity and mobilisation of natural killer cells, B cells and T_H_1-skewed T cells following treatment with Clevegen.^61,62^ It is evident that Clevegen treatment of these trial patients also targets sinusoidal endothelium in the liver, lymphatics and potentially tumour vasculature, if positive for Clever-1. Theoretically, it may impair some clearance functions of the liver. However, this is unlikely to cause any marked problems because of the abundance of different scavengers mediating clearance functions in the body. Blocking of Clever-1 on lymphatics, on the other hand, is expected to inhibit tumour spread, and on tumour vasculature, it may modify the quantity and quality of the incoming immune cells, as has been observed in mouse studies.^18^ Although Clever-1 targeting may have multiple consequences on different cell types, the final outcome is inhibition of tumour growth and spread in mouse models. Encouraging antitumour responses have also been observed in the MATINS trial patients, despite having an end-stage disease with high tumour burden and multiple lines (6–7) of previous treatments.^61^

## Association of Clever-1 with immunotherapy resistance

Advances in our understanding of tumour immunology have resulted in a new milestone for cancer therapy with the development of immune checkpoint inhibitors (ICIs). These therapeutic antibodies block negative immune regulatory checkpoints on T cells, thereby unleashing pre-existing antitumour immune responses. ICIs have drastically improved the survival of patients with advanced cancer in the past decade. Those that target CTLA-4, PD-1 or PD-L1, have been approved for the treatment of various cancer types, while others are still under clinical development.^63^ However, a large proportion of cancer patients are refractory to ICIs, or relapse despite these treatments by developing resistance mechanisms that are often associated with an immunosuppressive TME.^64^ In addition, clinicians still have very limited methods to discriminate responders from non-responding patients, justifying a wave of research into the fields of primary, adaptive and acquired resistance to immunotherapies.^64^ Importantly, TAMs, which have been reported to sustain local immunosuppression, are key targets to improve ICI efficacy.^65^ In fact, the ability of Clever-1-positive TAMs to suppress T_H_1 lymphocytes^38^ suggests that Clever-1 might be a potential target to overcome immunotherapy resistance. This notion is also supported by data showing that targeting Clever-1 in combination with anti-PD-1 in refractory (immunologically cold, such as colon [CT26] and triple-negative breast [4T1]) tumour models improves treatment efficacy.^47^

**Fig. 2:**
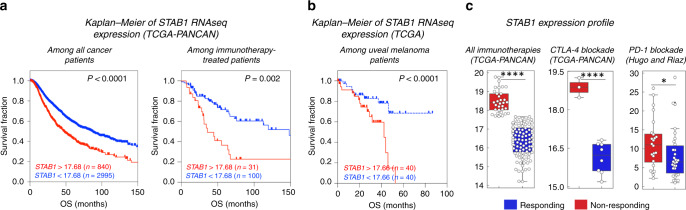
High levels of Clever-1 mRNA are associated with immunotherapy resistance. Kaplan–Meier survival plots of *STAB1* gene expression from **a** all patients in The Cancer Genome Atlas (TCGA) PANCAN (pan-cancer) cohort (left) or immunotherapy-filtered patients in the TCGA–PANCAN cohort (right), and **b** TCGA-primary uveal melanoma cohort. Normalised (HTSeq-FPKM-UQ) RNAseq datasets were downloaded from the Genomic Data Commons–TCGA portal using UCSC Xena (xena.ucsc.edu), including all clinical/phenotyping data information, and plotted using GraphPad Prism 8. The cut-off for *STAB1* expression was determined based on the best fit of each survival curve within the cohorts. Log-rank test was used to determine significant differences among the survival curves. **c** *STAB1* expression profile among responding and non-responding immunotherapy-treated cancer patients. Responding and non-responding patients from the TCGA–PANCAN cohort treated with all different immunotherapies or CTLA-4 alone were grouped according to the TCGA–PANCAN Kaplan–Meier survival curve. Responding and non-responding patients from Hugo and Riaz cohorts were grouped according to the clinical information available from both studies.^67,68^ An unpaired two-tailed *t* test was used for statistical analysis for normally distributed datasets (TCGA–PANCAN plots), and a Mann–Whitney two-tailed test was used for statistical analysis of not normally distributed datasets (Hugo and Riaz plot). *P < 0.05, **P < 0.01, ***P < 0.001, ****P < 0.0001. *P* < 0.05 was considered statistically significant. Statistical analyses were performed using GraphPad Prism 8. The data banks used to analyse Clever-1 associations are publicly available, the associations found have not been published previously.

Relatively few reports have been published addressing Clever-1 expression as a predictive or prognostic factor for patient outcome,^40,41,43,44^ and there are no reports of Clever-1 expression during ICI treatments; we, therefore, sought to investigate the significance of Clever-1 expression (at the mRNA level) in immunotherapy resistance, T-cell exclusion and dysfunction by extracting data from publicly available clinical datasets. Generally, a high expression of *STAB1* in cancer patients of The Cancer Genome Atlas pan-cancer cohort (*n* = 11768) is significantly associated with reduced survival, regardless of patient characteristics, tumour type, therapy or clinical manifestations (Fig. 2a, left). Furthermore, patients with high levels of *STAB1* who were treated with different immunotherapy regimens, including ICIs, show reduced overall survival (Fig. 2a, right). Interestingly, a similar survival pattern is observed in a small cohort of patients with uveal melanoma (Fig. 2b), one of the types of cancer that is most refractory to anti-PD-1 and anti-CTLA-4 blockade.^66^ Moreover, analysis of *STAB1* among all immunotherapy-treated patients from The Cancer Genome Atlas pan-cancer data, including anti-CTLA-4-treated cases and two PD-1 blockade cohorts,^67,68^ shows that *STAB1* expression is, indeed, significantly higher among the non-responding patients than the responders (Fig. 2c). This result might indicate that Clever-1 is a potential functional myeloid marker among the patients who respond poorly to different immunotherapy programmes.

**Fig. 3:**
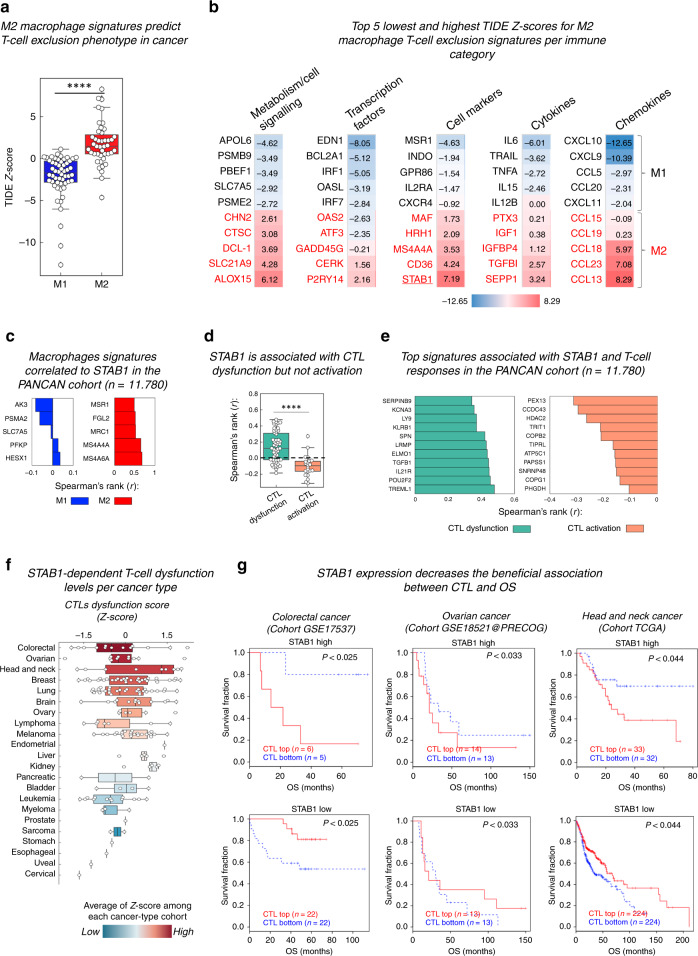
Clever-1-dependent T-cell exclusion and dysfunction in cancer. **a** T-cell exclusion scores computed in TIDE for different M1 and M2 gene signatures. A Mann–Whitney two-tailed test was used for statistical analysis of not normally distributed *Z* scores obtained for M1 and M2 signatures. **b** Representative heatmaps depicting the top five lowest and highest *Z*-score levels for different M1 and M2 signatures sorted by immune category. *STAB1* has the second highest score among macrophage signatures. **c** The top five lowest and highest Spearman correlation scores of *STAB1* with M1 and M2 macrophage signature genes described by Cassetta et al.^70^ **d** *STAB1* significantly correlates with signatures of T-cell dysfunction^69^ that predicts cancer immunotherapy response. **e** The top eleven positive and negative genes comprising T-cell signatures associated with CTL dysfunction and activation that correlate with *STAB1* within the PANCAN study, respectively. The normalised gene signatures were downloaded from the GDC portal using UCSC-Xena browser and aligned to *STAB1* expression across the PANCAN patients. Rank *r* scores were obtained by non‐parametric, two‐tailed, Spearman’s test, where *P* < 0.0001 was considered to indicate significant differences (**c**, **e**). **f** Box plots representing the distributional characteristics of the *Z* score of interaction terms from Cox–PH survival of each cancer type. Data were computed and extracted from a large number of clinical cancer datasets available in TIDE and plotted using Instant Clue software. **g** Representative Cox–PH survival regression curves of three cancer types (colorectal, ovarian and head and neck cancers) where *STAB1* expression significantly predicts the dysfunctional state of cytotoxic T lymphocytes (CTLs), defined by the decrease in beneficial association between CTL and overall survival (OS). Groups are divided in terms of high (top) and low (bottom) expression of CTL markers (*CD8A*, *CD8B*, *GZMA*, *GZMB* and *PRF1*). The TIDE portal applies to measure the interaction multivariate Cox–PH regression test to evaluate whether *STAB1* correlates with the dysfunction state of T cells as previously described.^69^ Briefly, if the effect of CTLs on survival outcome depends on the levels of *STAB1* (high and low), which is a typical case of interaction between two variables, the survival plots will show opposite results. Here, *STAB1* levels have significantly antagonistic interactions with CTL levels, indicating that a higher *STAB1* expression in the tumours will decrease the beneficial association between CTL and OS. TIDE Z scores for prediction of T-cell exclusion and dysfunction signatures were generated and extracted using the TIDE portal (tide.dfci.harvard.edu). The data banks used to analyse Clever-1 associations are publicly available, and the associations found have not been published previously.

To understand the reason for this correlation between Clever-1 positivity and resistance to immune checkpoint therapies, we used the online computational method TIDE (tumour immune dysfunction and exclusion) for a large number of clinical cancer datasets.^69^ TIDE models factors that mediate the induction of T-cell dysfunction in tumours with high infiltration of cytotoxic T lymphocytes (CTLs), as well as the prevention of T-cell infiltration in tumours with low levels of CTLs. As Clever-1 expression is commonly used as a marker of immunosuppressive (type 2) macrophages, we first verified that the human M2 gene signature^70^ can predict T-cell exclusion in the TIDE cohorts in comparison with the M1 gene signature (Fig. 3a). We further observed that *STAB1* has one of the highest *Z* scores (7.193) for the T-cell exclusion signature among the M1/M2 genes, specifically for the category of cell-surface markers, as displayed in an immune category heatmap, together with the chemokines CCL23 (7.079), CCL18 (5.97) and CCL13 (8.287) (Fig. 3b). The top genes having the most negative or lowest correlation with *STAB1* within the M1 signature genes and the top genes having the highest positive correlation with *STAB1* expression in the M2 signature genes across all cancer patients of the PANCAN study are shown (Fig. 3c). Altogether, these findings suggest that *STAB1* expression may be associated with myeloid mechanisms that impair T cells from reaching the tumour site, and thus, limiting the efficacy of ICI therapies.^71^ Thus, we investigated the correlation of *STAB1* expression across all cancer patients of the PANCAN study with recently described T-cell dysfunction and activation signatures.^69^ Strikingly, *STAB1* significantly correlated with signatures that predict CTL dysfunction, but negatively with those associated with CTL activation (Fig. 3d). The top positive and negative genes in the T-cell signatures correlating with *STAB1* are shown (Fig. 3e). Using the prediction score for CTL dysfunction in TIDE, we found that *STAB1* can significantly predict the dysfunctional state of CTLs in at least four different cancer types (colorectal, ovarian, head and neck and triple-negative breast cancer) (Fig. 3c). Interestingly, in the tumours that have high levels of *STAB1* mRNA, higher infiltration of CTLs is associated with decreased overall survival (upper Kaplan–Meier curves in Fig. 3d) whereas, in the low-*STAB1*-expressing tumours, higher CTL infiltration is associated with a more favourable overall survival (lower curves in Fig. 3d), suggesting that *STAB1* expression levels might indeed correlate with mechanisms that impair the antitumour effector functions of CD8^+^ T cells.

## Conclusions

**Fig. 4:**
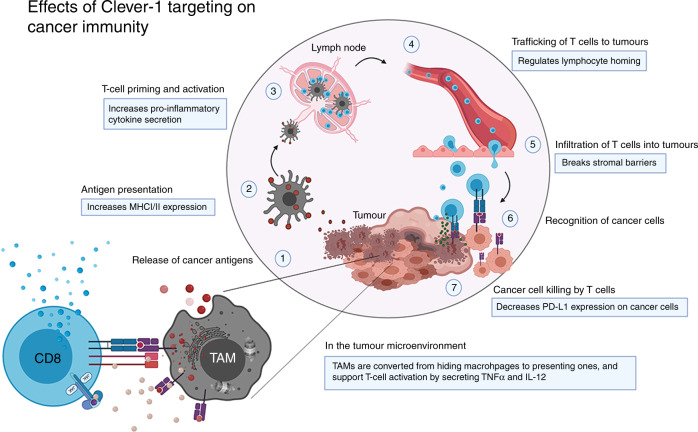
Clever-1 targeting has profound effects on boosting cancer immunity. Adopted from the cancer-immunity cycle by Chen and Mellman.^72^ Cancer-specific antigens are released from dying cells (1), which are taken up by antigen-presenting cells (2) to prime naive lymphocytes in the tumour-draining lymph nodes (3). Proper co-stimulation and an appropriate cytokine milieu activate T cells, which then traffic to the tumour site via the blood system (4) and infiltrate tumours (5). In the tumour, the T cells recognise their cognate antigen (6) on cancer cells and secrete granzyme B and other cytotoxic molecules to kill them (7). Blocking Clever-1 functions on macrophages and endothelial cells can boost several aspects in the cancer-immunity cycle. By increasing antigen presentation and pro-inflammatory cytokine secretion in tumour-associated macrophages (TAM), the tumour-infiltrating lymphocytes (CD8 T cells) can be re-activated to secrete interferon γ (IFNγ) and proliferate. Clever-1 blockade can also selectively inhibit trafficking of different leukocyte populations and cancer cells, which contributes to the different composition of the TME and reduced metastasis. Due to the conversion of macrophage functions by Clever-1 targeting, the stromal immunosuppressive barrier no longer prevents lymphocyte infiltration. Finally, and for as yet unknown reasons, immunotherapeutic targeting of Clever-1 reduces the expression of programmed death-ligand 1 (PD-L1) on cancer cells, making them more vulnerable to the activated immune attack. Modified from *Immunity* volume **39**, Chen, D.S. & Mellman, I. Oncology meets immunology: the cancer-immunity cycle, pages 1–10, Copyright (2013), with permission from Elsevier.

Clever-1 is a very versatile molecule and its broad impact on dampening antitumour immune responses has only just begun to be recognised. As such, both endothelial and macrophage subsets that express Clever-1 are important mediators of these actions, and their contribution may vary depending on the tumour type (Fig. 4). The dissection of novel mechanisms of cancer immunosuppression and immunotherapy resistance involving the biology of Clever-1-expressing TAMs aims to enable the translation of these research findings from bench to bedside, as is now in progress in the MATINS clinical trial. It is expected to provide solid ground for a new generation of Clever-1 inhibitors that could potentially act as alternative therapies to improve antitumour immune responses, as well as adjuvant strategies to prevent resistance that often occurs during the ICI therapies.

## Data Availability

All data presented in this review are publicly available.
